# Poor Mental Health during the COVD-19 Pandemic: Effect Modification by Age

**DOI:** 10.1177/0706743721994408

**Published:** 2021-02-16

**Authors:** Andrew Bulloch, Sara Zulyniak, Jeanne Williams, Janak Bajgai, Asmita Bhattarai, Ashley Dores, Aysha Lukmanji, Tiffany Pham, Kathryn Wiens, Scott B. Patten

**Affiliations:** 1 Department of Community Health Sciences, University of Calgary, Alberta, Canada

**Keywords:** virus, mental health, anxiety, epidemiology, pandemic

The COVID-19 disease, caused by a coronavirus known as SARS-CoV-2, was declared a pandemic by World Health Organization on March 11, 2020. The Canadian provinces and territories subsequently declared a state of public health emergency (e.g., Alberta on March 17 and Ontario on April 14). Public health disasters such as viral pandemics are known to result in long-lasting deleterious effects on mental health of both health-care workers and the general population. Regarding the general public, those exposed to SARS during 2003 in Hong Kong showed highly elevated symptoms of anxiety during the epidemic that declined to pre-epidemic levels after about 3 months.^1^ Similarly, the epidemic of MERS in Korea during 2015 was accompanied by high levels of anxiety and anger that declined substantially after 4 to 6 months.^2^ A recent report of Czech adults indicates increased levels of mental disorders, including anxiety and major depression, during the COVID-19 pandemic.^3^

We used data from the Statistics Canada’s probability-based survey known as *Canadian Perspectives Survey Series 2 (CPSS 2)—Monitoring the effects of COVID-19, 2020* (*N* = 4,600; conducted March 29 to April 3). We both extended previous work^4,5^ and added comparison data from a pre-COVID-19 referent sample. Specifically, we examined data from both available mental health variables: self-perceived mental health and symptoms of generalized anxiety disorder (GAD; as estimated by the GAD-7). To estimate the relative impact of COVID-19 on mental health, we used a referent sample from the probability-based Canadian Community Health Survey (CCHS) of 2017/2018 (*N* = 108,811). Use of the same variable (self-perceived mental health) from the 2 surveys provides a useful comparison of Canadian’s mental health before and during the pandemic. The GAD-7 has not been used in Canadian national surveys, so a similar approach is not possible for this measure. For the GAD-7, we used the customary cut-off score of 10, as indicated in a psychometric validation,^6^ to indicate moderate or severe symptoms, that is, as evidence of a need for further assessment or treatment. Self-perceived mental health was dichotomized as fair/poor or excellent/very good/good.

**Figure 1.**
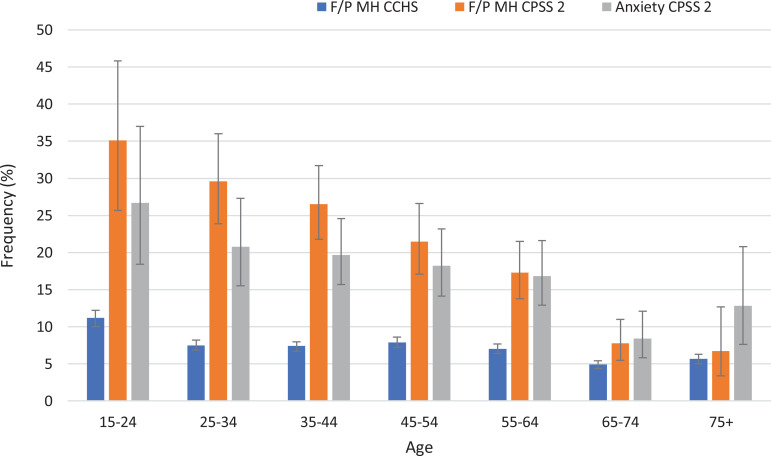
Estimated frequencies with associated 95% confidence intervals for poor/fair mental health in the referent group (“F/P MH CCHS”), and for poor/fair mental health and anxiety in the CPSS 2 survey (“F/P MH CPSS 2” and “anxiety CPSS 2”), respectively.

We calculated age-dependent frequencies of fair/poor self-perceived mental health and moderate/severe anxiety symptoms (Figure 1). Regarding the referent (CCHS 2017/2018) group, estimates for fair/poor self-perceived mental health among those in the 25 to 75+ age range showed a narrow prevalence range of 5% to 8%. For younger group (15 to 24 years), the corresponding estimate was about 11%. In contrast, data from the CPSS 2 showed a strong age dependence of both self-perceived fair/poor mental health and anxiety symptoms (for anxiety, this agrees with a preliminary estimate—4). Both measures of mental health were strongly elevated in 15- to 24-year-olds and declined with age. In the 65+ age range, the frequency of both measures during the pandemic resembled that of the referent, in sharp distinction in those under the age of 65. The point prevalence estimates for both measures were remarkably close. Each of these measures decreased steadily in near parallel fashion from 15-24 to 55-64 age groups and subsequently dropped more steeply and intersects with the referent group of 65 to 74.

Of those that reported fair/poor mental health, 49.4% (95% CI, 43.3% to 55.5%) had elevated GAD-7 scores. Of those who reported excellent/very good/good self-perceived mental health, elevated GAD-7 scores were seen only in 9.4% (95% CI, 7.7% to 11.3%). The high prevalence of anxiety symptoms in those reporting fair or poor mental health is notable.

In conclusion, the COVID-19 pandemic is associated with a substantial increase of poor mental health as estimated by both self-reported mental health and assessment of GAD symptoms, but is only evident in those under age 65. A report from early in the pandemic indicated that risk factors for fair/poor mental health at this stage included having a physical condition and family stress.^5^ Further research is needed to provide (a) a long-term perspective of mental health during and after the COVID-19 pandemic and (b) to relate elevated symptoms to the occurrence of diagnosed mental disorders. Our findings indicate a considerable burden to the mental health services and the need for innovative approaches such as Employment Assistance Programs support for psychotherapy.

Perceived mental health is difficult to interpret as it relates to perceptions rather than symptoms. The results presented here clarify that such perceptions are strongly related to symptom levels of anxiety and support the use of this measure in future studies.

A high prevalence of negative perceptions of mental health in the *Canadian Perspectives Survey* is difficult to interpret in isolation, given the absence of a reference group. Fortunately, the CCHS provides a referent. A striking change in the pattern of fair or poor mental health is seen, with a larger worsening of perceived mental health in younger respondents with no effect on 65+ respondents. It may be that the social disruption of the public health response, which may disproportionally affect youth, is a greater determinant of anxiety symptoms than medical fears. The latter are probably more prominent in the elderly.
